# Near‐Infrared Spectroscopy as a Resource for Assessing the Rest and Activation of Pelvic Floor Muscles in Young Adult Women: A Cross‐Sectional Study

**DOI:** 10.1002/jbio.202500255

**Published:** 2025-10-22

**Authors:** Iasmin Pereira Cabral Miranda, Marcos Venicius Bentes do Nascimento, Pablo Fabiano Moura das Neves, Maria Clara Pinheiro do Nascimento, Fernanda Caroline de Jesus Viana, Mayara Carolina Jorge Moraes, Emili Beatriz Chaves de Brito, Fabiane Yasmin de Miranda Lobato, Rayanne Mesquita Bendelack, Elizabeth Alves Ferreira, Givago da Silva Souza, João Simão de Melo Neto

**Affiliations:** ^1^ Universidade Federal do Pará (UFPA) Belém PA Brazil; ^2^ Fundação Santa Casa de Misericórdia do Pará (FSCMP) Belém PA Brazil; ^3^ Instituto de Estudos Superiores da Amazônia Belém PA Brazil; ^4^ Universidade da Amazônia (UNAMA) Belém PA Brazil; ^5^ Centro Universitário da Amazônia (UNIESAMAZ) Belém PA Brazil; ^6^ Universidade de São Paulo São Paulo SP Brazil

**Keywords:** near‐infrared spectroscopy, pelvic floor muscles, woman's health

## Abstract

This study evaluated the potential of near‐infrared spectroscopy (NIRS) as a noninvasive tool to assess pelvic floor muscle oxygenation in young adult women. Thirty‐eight participants were divided into a control group (rest) and an activation group (phasic and sustained contractions). Measurements were obtained with the Humon Hex device placed on the skin overlying the bulbospongiosus and superficial transverse muscles. Muscle activation, especially during maximum sustained voluntary contraction, resulted in significant oxygenation changes, with greater variability in the activation group. The bulbospongiosus showed higher sensitivity, whereas the transverse muscle presented more stable responses. Despite limitations related to sensor adaptation and signal stability, NIRS detected differences in metabolic demands between rest and activation when placed on the skin surface. These findings support NIRS as a potential and comfortable alternative to transvaginal intracavitary techniques, contributing to objective evaluation, prevention, and rehabilitation strategies in women's pelvic health.

## Graphical abstract

This study shows that near‐infrared spectroscopy (NIRS) is capable of identifying changes in oxygenation of the pelvic floor muscles during rest and contraction, offering a promising noninvasive alternative for clinical assessment and research in women's health.

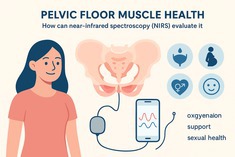

**Abbreviations**
AG: activation group
BMI: body mass index
CG: control group
IQR: interquartile range
MVSC: maximum sustained voluntary contraction
NIRS: near‐infrared spectroscopy
PC: phasic contraction

## 1 Introduction

The pelvic floor is a set of structures that are involved in supporting abdominal and pelvic organs, offering resistance to increased intra‐abdominal pressure and contributing to lumbar stabilization, urinary and fecal emptying, maintenance of vaginal pressure, and sexual health [1, 2, 3]. Dysfunctions related to these important structures may be experienced by one in four women aged 20 years or over during their lifetime [4]. The scale of the public health problem, as well as the psychological, physical, and economic burdens on women, requires special attention to the condition, especially in relation to treatment, which usually includes pelvic floor muscle training, with strong scientific evidence regarding the benefits for rehabilitation [5].

In this context, an objective assessment of the pelvic floor muscles is essential not only to guide treatment planning but also to monitor the effects of therapeutic interventions. This is because the adequate prescription of training intensity and dosage relies on precise information about the muscles' ability to generate contraction, sustain it, and return to relaxation, ensuring both the safety and effectiveness of the intervention [3, 6, 7, 8, 9, 10].

The researchers of this study are particularly concerned with assessment practices in the local context. Health care is provided at a reference hospital for maternal and child health in the northern region of Brazil. In this location, we frequently treat patients as follows: minors; those with intact hymens; high‐risk pregnant women; victims of sexual abuse; muscular hyperactivity; or those in whom voluntary muscle contraction is not observed.

In the literature, the most common methods for assessing pelvic floor muscles in clinical practice include digital palpation, perineometry, electromyography, manometry, and imaging techniques such as ultrasound and MRI. However, many of these are described as subjective, with low sensitivity and reproducibility, in addition to presenting challenges related to patient adherence and, in some cases, contraindications—particularly at the beginning of treatment, when external evaluation may be feasible [7, 11].

The assessment and training of the pelvic floor muscles can be constantly problematic because of the absence of a technique considered the gold standard, as well as standardized methodologies to quantify measures of function, strength, resistance, and neuromuscular activation in an objective, reliable, and easy‐to‐use manner [12, 13].

Recognizing this practical and scientific deficiency, current measures for evaluating these muscles through measurements of muscle oxygenation kinetics are necessary. Near‐infrared spectroscopy offers a non‐invasive approach to indirectly quantify muscle strength by monitoring changes in local oxygenation and hemodynamics during contraction and relaxation. When a muscle is activated, increased metabolic demand leads to variations in oxygen consumption and blood volume, which can be detected by NIRS sensors in real time. The magnitude and pattern of these hemodynamic responses are correlated with the intensity of contraction, allowing NIRS to serve as an objective indicator of muscle strength and endurance. In the context of pelvic floor muscles, this technique may provide a valuable alternative to conventional methods, enabling a more precise evaluation of contractile capacity and functional performance [14]. As in sports medicine, the response of skeletal muscle tissue to exercise is often performed by investigating measures related to the intensity of effort during muscular activation, such as maximum oxygen consumption [15].

A growing method for assessing muscle oxygenation in different sports and athletic training interventions is surface near‐infrared spectroscopy [14, 15, 16]. The reliability of its measurements in real‐time monitoring of both health and pathological states and the associations of these with physical fitness parameters has been frequently described in the literature and highlights a vast field for new applications with great potential for transforming current clinical practices [17, 18, 19, 20].

The use of this technology has been indicated for acquiring information on the workload capacity of muscles (oxygen extraction) and resistance to fatigue, with significant advantages in terms of accessibility, portability, suitability for use in remote conditions and poor infrastructure, with reports of greater tolerance by patients, in addition to corroborating the trend toward remote monitoring and personalization of therapies on the basis of muscle training [21, 22, 23, 24].

Although its use has been previously described by Macnab et al. [21, 25] in an intracavitary manner in the evaluation of pelvic floor muscle (PFM) activation, studies that explore the capacity of this technology in acquiring superficial information about the muscles of this region have not yet been located and hold the potential for important social, financial, and scientific impact.

The main advantages of NIRS are its non‐invasive nature, high repeatability, and good patient acceptance. In addition, its ability to measure tissue oxygenation and blood volume makes it a valuable tool for deepening pathophysiological and diagnostic knowledge, monitoring the evolution of functional deficiencies, identifying correlations with clinical symptoms, and evaluating therapeutic interventions [24], which motivated our interest in exploring its applicability in women's health care.

For this reason, we raised the following question: “Do oxygenation variables obtained by near‐infrared spectroscopy equipment, used superficially on the pelvic floor muscles of young adult women, detect changes during the states of muscle contraction and rest?”

Therefore, the aim of this study was to investigate the behavior of muscle oxygenation variables obtained by a surface near‐infrared spectroscopy device during rest and activation of the pelvic floor muscles in young adult women. Our hypothesis is that there is a reduction in muscle oxygenation levels during activation of the pelvic floor muscles in young adult women. Thus, this study and future investigations in this field are valuable for the evolution of scientific and clinical practices in women's health care.

## 2 Methods

### 2.1 Ethical Aspects

The study was carried out after approval by the Ethics and Research Committee of the Santa Casa de Misericórdia do Pará Foundation (FSCMP) under opinion 6.666.755 and written signature of the Informed Consent Form (ICF). The subjects were studied in accordance with the Declaration of Helsinki and the standards for research involving human beings (Res. 466/12 and Res. 510/16 of the National Health Council).

### 2.2 Study Design

This was a cross‐sectional observational study of randomized, blinded, analytical, descriptive, and inferential types.

### 2.3 Setting and Period of Study

The samples were collected at a public maternal and child hospital in Belém, PA. The collection period was from 26/02/2024 to 30/09/2024.

### 2.4 Population

The participants were women aged 18 –35 years and were considered young adults.

### 2.5 Sampling

Sampling is non‐probabilistic of the convenience type.

### 2.6 Sample Size

The sample calculation was carried out via a statistical approach based on the formula for comparing the means of two independent groups (*n* = 2(*σ* ^2^)(z1 − *α*/2 + z1 − *β*)^2^/Δ^2^)) and was conducted to ensure statistical rigor in the analysis of differences in oxygenation variables between homogeneous groups via near‐infrared spectroscopy on the basis of information extracted from the literature [14, 16, 17, 25]. Classical statistical parameters were considered, with a significance level (*α* = 0.05) and statistical power (1 − *β* = 0.80). In addition, the expected effect size (Δ) was set at 10%, which represents the smallest clinically relevant difference between the groups studied. The standard deviation (*σ*), especially that reported by Miranda‐Fuentes et al. [16], indicated a variability of 5.76% for the mean oxygen saturation at rest. This parameter was selected as a conservative estimate, reflecting the variability expected in muscle oxygen measurements. In addition, we used critical values for the z scores, with z1 − *α*/2 = 1.96z_{1 − *α*/2} = 1.96z1 − *α*/2 = 1.96 for a 95% bilateral confidence interval and z1 − *β* = 0.84z_{1 − *β*} = 0.84z1 − *β* = 0.84 to guarantee 80% statistical power.

The formula used to determine the necessary sample size was applied (*n* = 2(5.76^2^) (1.96 + 0.84)^2^/10^2^), resulting in *n* = 5.20. The minimum rounded value for each group was 6 participants, totaling a minimum desirable sample of 12 participants in the study. However, the sample in this study included 38 participants.

### 2.7 Eligibility Criteria

The study included women aged between 18 and 35 who agreed to take part by signing an informed consent form. Women with the following characteristics were excluded from the study: allergy to the latex of the glove, infectious conditions, pregnancy, inability to perform voluntary contraction of the PFM, known dysfunction related to the pelvic floor, current menstruation or the presence of recent gynecological bleeding, childbirth or gynecological surgery in the last 6 months. In addition, those who refused or requested that the research procedures be interrupted were excluded, as were those who chose not to provide all their data, which was characterized as incomplete data.

### 2.8 Data Collection and Variables

A form developed by the authors was used to collect data from all patients. The variables collected to characterize the sample were social (age and declared race/color), anthropometric (current weight, height, and body mass index), urogynecological (sexually active in the last 6 months; pain during intercourse; menstrual cycle; contraceptive use; urinary tenesmus; dysuria; urinary loss on exertion; urinary loss during urgency), obstetric (pregnancies; abortion; vaginal delivery; surgical delivery), proctologic (fecal tenesmus; evacuation effort; fecal incontinence), dominant laterality, and level of physical activity. In addition, the level of muscle oxygenation was obtained.

Oxygenation variables (mean oxygenation, oxygenation amplitude, and standard deviation) were obtained using the Humon Hex device (Dynometrics Inc., Boston, MA, USA), a portable near‐infrared spectroscopy (NIRS) sensor specifically designed to monitor muscle oxygenation during exercise and validated for muscle studies [16]. The device measures 60.5 × 57 × 13.8 mm, weighs 32 g, and has a curved medical‐grade plastic case that facilitates stable contact with the skin surface. It operates with dual wavelength emitters (680 nm and 850 nm) and three inter‐optode distances (11, 22, and 33 mm), allowing for spatially resolved geometry to estimate local tissue absorption and scattering properties. Data are transmitted via Bluetooth and ANT+ to a dedicated mobile app (MoxZones Solutions, Dynometrics Inc.), which provides real‐time visualization of muscle oxygen saturation (SmO_2_) through color‐coded training zones and post‐exercise analysis. Although its algorithms have shown strong agreement with a bench system (96% accuracy and 3.4% RMSE in incremental cycling tests) and have been able to predict the lactate threshold within ±21 W, the signal processing methods are proprietary and not disclosed by the manufacturer. This is a significant limitation, as it restricts independent verification of the mathematical models and may influence the reproducibility of the derived hemodynamic parameters. The raw data from the experimental sessions were exported in spreadsheet format via the MoxZones platform (https://moxzones.com/) [26, 27, 28, 29, 30].

### 2.9 Data Collection

After signing the informed consent form and collecting initial information via the evaluation form, the volunteers were instructed to undergo a physical examination of the pelvic floor muscles, which was conducted by a trained researcher. The order of the assessments as to group, control (CG) and activation (AG), was randomized and blinded to the participants. All the assessment stages were carried out on the same day, with a 10‐min interval between them [31].

Before the physical examination, the near‐infrared spectroscopy equipment was sanitized with Riohex 2% chlorhexidine enzyme detergent. The equipment was exposed to the product for 10 min, rubbed in a circular motion on the surfaces by the researcher's gloved hands, and then carefully dried with paper towels for packaging in sealed, disposable plastic packaging.

The volunteers were instructed to undress from the waist down behind the screen, wear an individual, disposable protective apron, and then help climb onto the stretcher to lie down in dorsal decubitus with the back inclined at 30°, legs abducted, knees semiflexed, and feet supported. The pelvis was kept in a previously determined neutral position on the stretcher, with no need to change the ambient light for collection.

The participants were then instructed by a trained physiotherapist to activate the pelvic floor muscles with the command “squeeze the vagina and anus, as if to hold in the pee and poop.” The correct contraction was identified by the elevation in and around the urethra, vagina, and rectum, without any visible movement of the pelvis or lower extremities [32]. 60 s after the visual identification of pelvic floor elevation, the physical examination steps were carried out according to randomization.

The near‐infrared spectroscopy (NIRS) emitter/detector array was positioned in the perineal region, covered by a single layer of disposable plastic film (replaced after each use), in direct contact with the pelvic floor muscles and adjacent tissues, specifically in the anatomical location corresponding to the bulbospongiosus or superficial transverse, according to the desired collection point. The positioning was defined by manual palpation and followed a reference similar to that adopted in noninvasive electromyography protocols, which are widely used in pelvic floor assessment [33, 34, 35, 36, 37, 38].

All procedures were conducted by a trained physical therapist specializing in women's health, who manually applied the near‐infrared spectroscopy (NIRS) device, keeping the light sensor (on/off) oriented toward the center of the perineum and covered by a single layer of disposable plastic film. The application was performed with light and uniform pressure, sufficient to ensure adequate contact with the skin surface without causing discomfort. Despite these standardization strategies, there may have been influences on the stability of the sensor, constituting a methodological limitation to be considered in the interpretation of the results and overcome in future studies.

To better represent oxygenation data in muscle activation, data were collected from the patient's dominant side, since previous studies with NIRS devices have shown differences between the left and right sides, consistent with the laterality of the individual evaluated, both in skeletal muscle [16] and specifically in the pelvic region in intracavitary evaluation [25]. For ambidextrous participants, the assessment was performed arbitrarily on the left side of the body.

**FIGURE 1:**
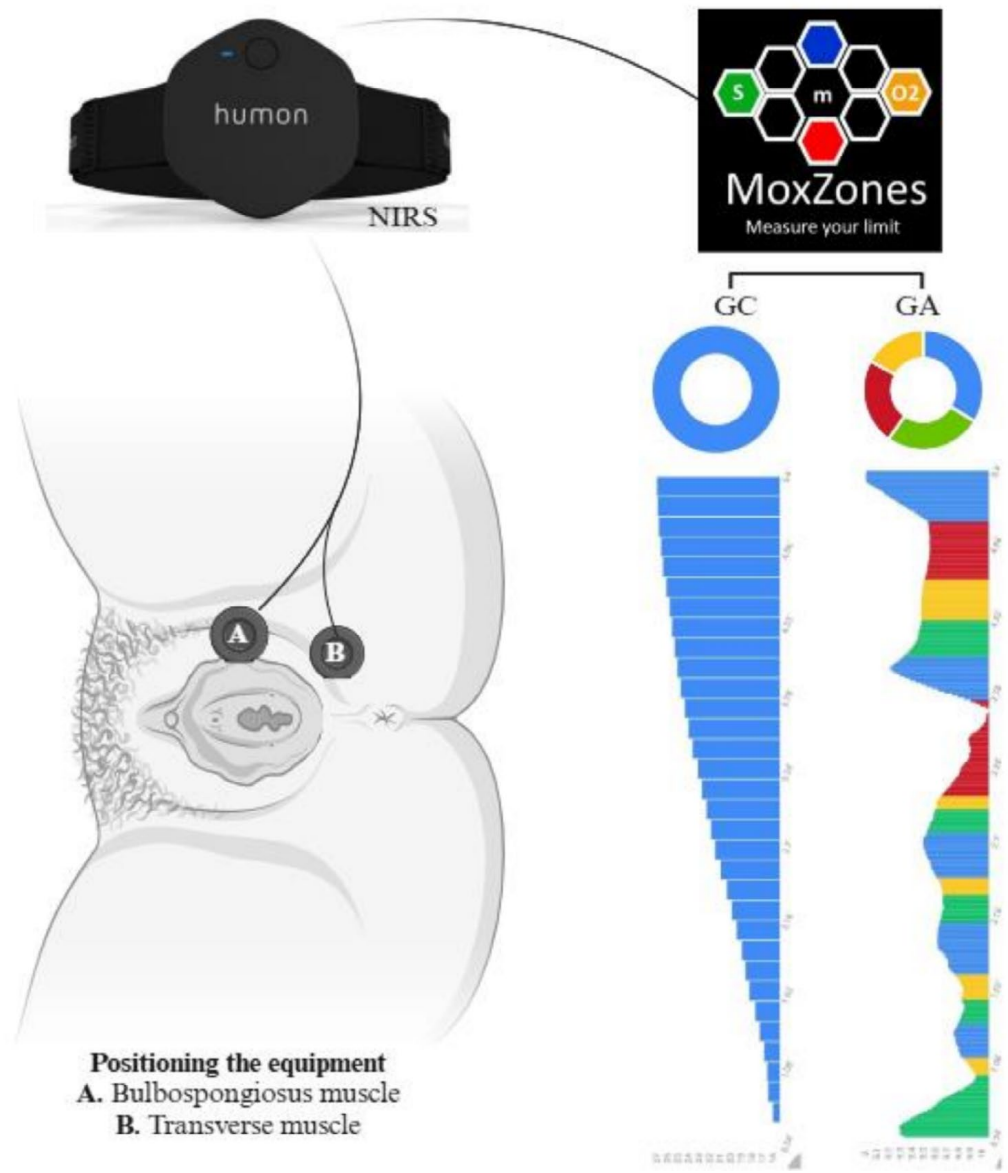
Schematic of the collection of surface near‐infrared spectroscopy of the pelvic floor muscles in young adult women. CG: AG: activation group; control group; NIRS: near‐infrared spectroscopy. Created by BioRender.com.

Like in the study by Macnab, Stothers, and Deegan [25], the raw data were captured at 10 Hz, and the data stream was also offset to an arbitrary zero; in this case, the initial 60 s were used to collect the concentrations of viable resting chromophores for later analysis. A representation of the collection is shown in Figure 1.

The participants in the CG were asked to maintain muscle rest during a 5 min (300 s) interval to collect information on each muscle (bulbospongiosus and superficial transverse), with a 10 min interval between them. The AG, on the other hand, performs a standardized sequence of exercises after an initial rest of 120 s, similar to previous studies [21, 25] in which surface near‐infrared spectroscopy of the pelvic floor muscles was used.

The sequence of exercises was as follows: 3 maximal voluntary phasic contractions (PCs) starting at 2 min (120–180 s), with a maximum interval of 15 s between them, followed by a recovery period. At the 3 min mark (180–300 s), they begin to perform a verbally stimulated Maximum Voluntary Sustained Contraction (MVSC) to be maintained for 10 s, followed by a verbal stimulus to rest the muscles. Data from 60 to 240 s were used for analysis.

The command “squeeze the vagina and anus as if you were going to hold in the pee and the poo, as hard as you can for 3 quick times in a row” was used in a standardized way, whereas the command “squeeze the vagina and anus as if you were going to hold in the pee and the poo, as hard as you can for as long as you can” was used to collect data on MVSC.

After evaluation, the film wrapping was discarded, and the equipment was sanitized again with Riohex 2% chlorhexidine enzyme detergent so that it could be properly packaged for evaluation by a new volunteer.

The raw data, after being extracted from the MoxZones platform, was tabulated in a Microsoft Excel spreadsheet with the help of an open‐source Python tool (Streamlit), which allowed the researchers to create an interactive web application to visualize the data.

To analyze oxygenation in the resting and muscle activation states, we used the variables mean oxygenation, oxygenation amplitude, and standard deviation. These variables were chosen as suitable representatives to characterize the behavior of the data in terms of variability and dispersion [39].

**FIGURE 2:**
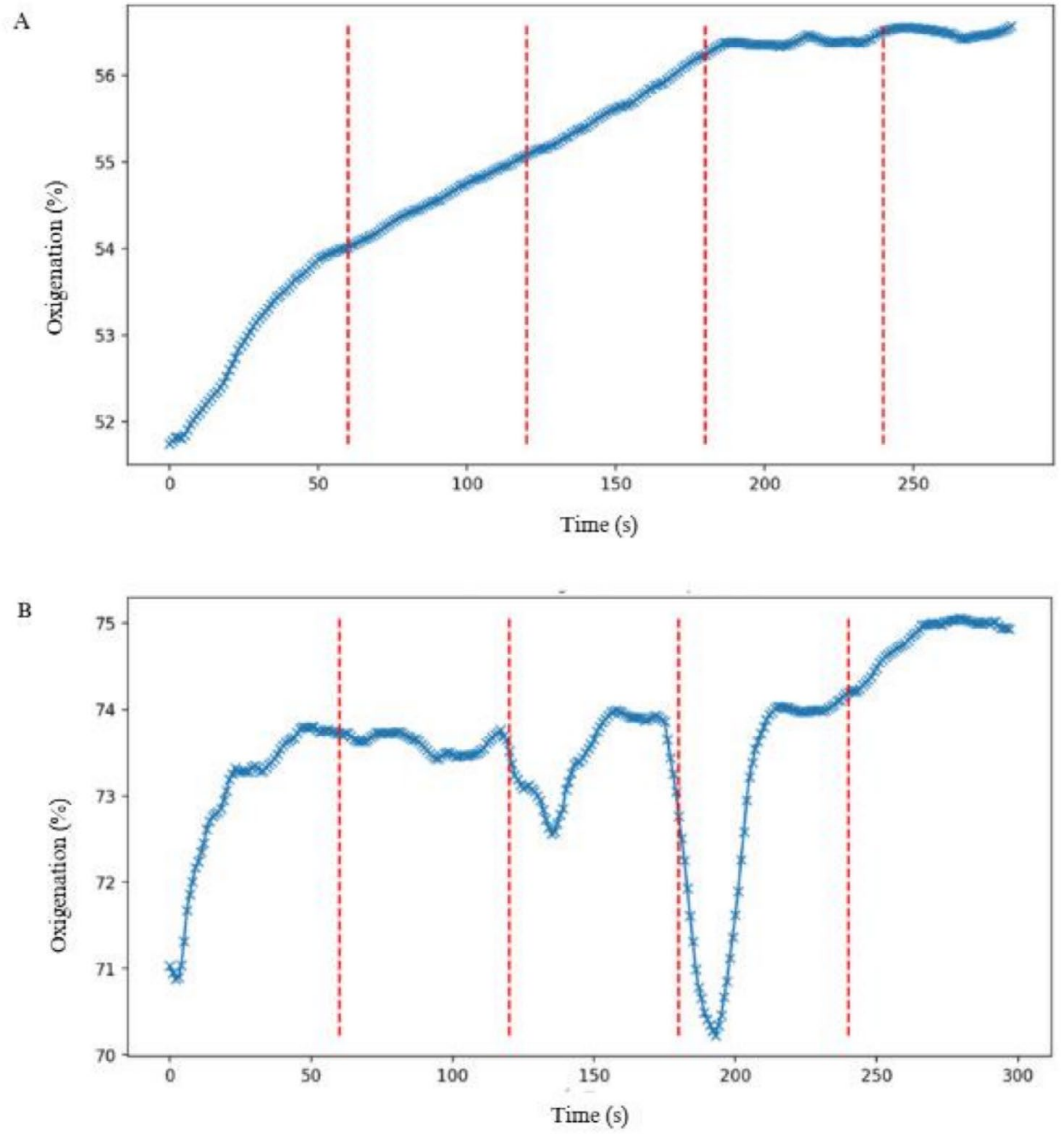
Representation of the behavior of oxygenation curves captured by infrared spectroscopy placed on the skin surface over the muscles of interest, observed consistently in the control (A) and activation (B) groups of the pelvic floor muscles in young adult women.

The variables were analyzed according to the intervals of the evaluation protocol (Figure 2) in which CG (A) performed muscle rest from 60 to 240 s and the AG (B) was divided into the following intervals: 60 –120 s: rest, 120–180 s: phasic contraction (PC), 180–240 s: MSVC. For methodological control, the intervals of 0 –60 s were disregarded for both groups in order to stabilize the signal, and 240–300 s were disregarded due to a lack of physiological interest in the present study.

In the end, the volunteers who wished received a conventional evaluation via the PERFECT scheme [40] and health education on PFM via graphic images, received feedback on the results of the physical evaluation and the availability of a professional specializing in Physiotherapy in Women's Health to answer any questions.

### 2.10 Bias

The study design was susceptible to recall bias, which we tried to minimize by randomizing the volunteers and blinding them.

### 2.11 Statistical Analysis

To make the appropriate intra‐ and intergroup comparisons, statistical tests were used, such as the Shapiro–Wilk normality test and nonparametric tests due to the distribution characteristics of the variables (Mann‐Whitney and Friedman), as well as effect size calculations (*r*) for each test, using the formulas *r* = (Mann–Whitney) and *r* = (Friedman), considering small (*r* = 0.10–0.29), moderate (*r* = 0.30–0.49), and large (*r* ≥ 0.50) [39]. The analyses were carried out using *RStudio version 4.4.2* and *JAMOV version 2.3.28.0*.

## 3 Results

The assessment was carried out on 40 volunteers. The CG had sample loss due to the instability of the internet network, which meant that the data of two volunteers was compromised. A total of 38 women were included in this study: 18 in the CG and 20 in the AG.

The sociodemographic, anthropometric, urogynecological, obstetric, and proctological profiles and the level of physical activity of the volunteers in the different groups are shown in Tables 1 and 2. All the volunteers had right‐hand dominant laterality.

**TABLE 1:** Characterization of the participants' social and anthropometric data (*n* = 38).

				Measures of central tendency		Measures of dispersion			Shapiro–Wilk	
	Group	*N*	Omitted	Mean	Median	Standard deviation	Minimum	Maximum	*W*	*p*
Age (years)	Activation	20	0	25.45	25.00	3.73	19	32	0.957	0.484
	Control	18	0	25.94	26.00	4.70	18	33	0.923	0.148
Weight	Activation	20	0	66.62	68.00	12.60	46.00	99.90	0.926	0.130
	Control	18	0	68.03	67.55	13.84	46.00	95.00	0.944	0.342
Height	Activation	20	0	1.64	1.65	0.04	1.57	1.72	0.970	0.748
	Control	18	0	1.63	1.63	0.06	1.50	1.77	0.971	0.824
BMI	Activation	20	0	24.71	25.15	4.37	17.97	36.69	0.932	0.172
	Control	18	0	25.86	24.70	5.50	17.97	38.67	0.903	0.065

Abbreviation: BMI: body mass index.

**TABLE 2:** Characterization of the social, urogynecological, obstetric, and proctological variables and level of physical activity of the sample (*n* = 38).

Variables	Category	Group	Frequency absolute	Relative frequency (%)
Declared race/skin color	White	Activation	11	28.9
		Control	6	15.8
	Brown	Activation	9	23.7
		Control	10	26.3
	Black	Activation	0	0.0
		Control	2	5.3
Daily physical activity	I walk or stand a lot during the day, but I don't carry or lift things often	Activation	3	7.9
		Control	6	15.8
	I carry light weights/climb stairs frequently/take regular exercise	Activation	11	28.9
		Control	7	18.4
	I do heavy work or heavy exercise regularly	Activation	3	7.9
		Control	2	5.3
	I don't walk much during the day	Activation	0	0.0
		Control	1	2.6
	I spend most of the day sitting down	Activation	3	7.9
		Control	2	5.3
Sexually active	No	Activation	7	18.4
		Control	5	13.2
	Yes	Activation	13	34.2
		Control	13	34.2
Pain during intercourse	0–3	Activation	13	34.2
		Control	16	42.1
	4–6	Activation	1	2.6
		Control	0	0.0
	7–10	Activation	6	15.7
		Control	2	5.2
Menstrual cycle	Non‐existent	Activation	1	2.6
		Control	2	5.3
	Irregular	Activation	7	18.4
		Control	5	13.2
	Regular	Activation	12	31.6
		Control	11	28.9
Contraceptive use	No	Activation	15	39.5
		Control	10	23.3
	Yes	Activation	5	13.2
		Control	8	21.1
Urinary tenesmus	No	Activation	17	44.7
		Control	13	34.2
	Yes	Activation	3	7.9
		Control	5	13.2
Dysuria	No	Activation	20	52.6
		Control	16	42.1
	Yes	Activation	0	0.0
		Control	2	5.3
Urinary loss on exertion	Never happened	Activation	15	39.5
		Control	13	34.2
	Yes, it happens occasionally	Activation	5	13.2
		Control	5	13.2
Urinary loss during urgency	Never happened	Activation	14	36.8
		Control	14	36.8
	Yes, it happens occasionally	Activation	6	13.8
		Control	4	10.5
Fecal Tenesmus	No	Activation	13	34.2
		Control	12	31.6
	Yes	Activation	7	18.4
		Control	6	15.8
Evacuation effort	No	Activation	6	15.8
		Control	8	21.1
	Yes, eventually	Activation	13	34.2
		Control	9	23.7
	Yes, always	Activation	1	2.6
		Control	1	2.6
Fecal incontinence	No	Activation	19	50.0
		Control	17	44.7
	Yes, eventually	Activation	1	2.6
		Control	1	2.6
Pregnancy	0	Activation	19	50.0
		Control	14	36.8
	1	Activation	1	2.6
		Control	3	7.9
	2	Activation	0	0.0
		Control	1	2.6
Abortion	0	Activation	20	52.6
		Control	17	44.7
	1	Activation	0	0.0
		Control	1	2.6
Vaginal delivery	0	Activation	20	52.6
		Control	17	44.7
	1	Activation	0	0.0
		Control	1	2.6
Surgical delivery	0	Activation	19	50.0
		Control	15	39.5
	1	Activation	1	2.6
		Control	3	7.9

Table 3 shows the intra‐group comparisons of each muscle oxygenation variable across the different intervals of the CG and AG. For the CG, the bulbospongiosus muscle presented an increase in the mean value of oxygen saturation during the assessment (*p* = 0.0005, Friedman test), which was more pronounced during MVSC (*p* < 0.05, Bonferroni test). For the transverse muscle, this group experienced equal results (*p* < 0.0001, Friedman test), with a progressive increase in the mean during rest in the CG in relation to the different types of contraction (PC and MVSC) (*p* < 0.05, Bonferroni test). With respect to the amplitude variable, the CG showed a reduction in the amplitude of the time series for both muscles during the assessment: bulbospongiosus (*p* = 0.033, Friedman test) and transverse (*p* = 0.027, Friedman test). However, when the post‐test data were analyzed, there was a significant reduction in PC for the bulbospongiosus muscle (*p* < 0.05, Bonferroni test) and MVSC for the transverse muscle (*p* < 0.05, Bonferroni test). Finally, the transverse muscle of the AG also showed an increase in the mean value of oxygen saturation during the assessment (*p* = 0.023, Friedman test).

**TABLE 3:** Comparisons of the oxygen saturation variables in the different intervals of each experimental group are presented as medians and interquartile ranges.

	Rest	PC	MSVC	*p*	Effect size (*r*)
Bulbospongiosus muscle—CG					
Mean	49.23 (15.86)^a^	50.27 (13.49)	50.99 (12.56)^b^	0.0005*	0.490
Range	1.21 (1.23)^a^	0.85 (0.63)^b^	0.64 (1.21)	0.0335*	0.2702
Standard deviation	0.3 (0.38)	0.21 (0.17)	0.18 (0.35)	0.0755	0.2396
Bulbospongiosus muscle—AG					
Mean	44.53 (29.35)	43.14 (31.99)	42.98 (30.92)	0.2466	0.1424
Range	1.38 (0.93)	1.21 (1.1)	1.94 (1.5)	0.1378	0.2423
Standard deviation	0.36 (0.32)	0.35 (0.33)	0.52 (0.39)	0.3442	0.1654
Transverse muscle—CG					
Mean	60.23 (18.76)^a^	62.64 (17.2)^b^	63.91 (16.16)^b^	< 0.0001*	0.5415
Range	1.57 (1.52)^a^	1.01 (0.69)	0.89 (0.94)^b^	0.0171*	0.2746
Standard deviation	0.45 (0.44)	0.28 (0.29)	0.31 (0.20)	0.0617	0.2303
Transverse muscle—AG					
Mean	62.56 (18.58)	63.38 (17.68)	65.18 (17.14)	0.0235*	0.2887
Range	1.41 (1.39)	1.24 (0.73)	1.46 (1.86)	0.3902	0.1446
Standard deviation	0.43 (0.6)	0.40 (0.24)	0.40 (0.51)	0.5698	0.1118

Abbreviations: AG: activation group; CG: Control group; MSVC: maximum sustained voluntary contraction; PC: phasic contraction.

* Statistical significance (*p* < 0.05) Friedman test. ^a,b^ *p* < 0.05, Bonferroni post‐test.

Tables 4 and 5 show the comparison of the inter‐group metrics (CG vs. AG) during the same intervals. There was no statistically significant difference between the groups for both muscles in the interval without muscle activation, represented by the rest interval (60–120 s) (*p* > 0.05, Mann–Whitney test). In terms of the metrics at the other intervals, there was an increase in oxygenation (*p* < 0.05, Mann–Whitney test) in the AG when the bulbospongiosus muscle (Table 4) was assessed at intervals between 120 and 180 s and between 180 and 240 s, and in the transverse muscle (Table 5) in the interval 180 and 240 s, represented in both muscles by the oxygenation amplitude and standard deviation.

**TABLE 4:** Comparisons of the oxygenation variables of the bulbospongiosus muscle at the same intervals between the experimental groups are presented as medians and interquartile ranges.

	CG	AG	*p*	Effect size (*r*)
60–120 s (Rest)				
Mean	49.23 (15.86)	44.53 (29.35)	0.8237	−0.0371
Range	1.28 (0.89)	1.36 (1.92)	0.2494	−0.2036
Standard deviation	0.3 (0.37)	0.36 (0.31)	0.5688	−0.1007
120–180 s (PC)				
Mean	50.27 (13.49)	43.14 (31.99)	0.7745	−0.0478
Range	0.92 (0.62)	1.27 (1.5)	0.0572*	−0.3415
Standard deviation	0.23 (0.17)	0.36 (0.47)	0.0453*	−0.3595
180–240 s (MSVC)				
Mean	50.99 (12.56)	42.98 (30.93)	0.6105	−0.0849
Range	0.91 (1.11)	2.06 (1.66)	0.0155*	−0.4349
Standard deviation	0.28 (0.32)	0.54 (0.49)	0.0135*	−0.4365

Abbreviations: AG: activation group; CG: Control group; MSVC: maximum sustained voluntary contraction; PC: phasic contraction.

* *p* < 0.05, Mann–Whitney test.

**TABLE 5:** Comparisons of the oxygenation variables of the transverse muscle at the same intervals between the experimental groups are presented as medians and interquartile ranges.

	CG	AG	*p*	Effect size (*r*)
60–120 s (Rest)				
Mean	60.24 (18.76)	62.56 (18.58)	0.7146	−0.0601
Range	1.57 (1.65)	1.41 (1.57)	0.7171	−0.0621
Standard deviation	0.45 (0.5)	0.48 (0.56)	0.836	−0.0355
120–180 s (PC)				
Mean	62.65 (17.2)	63.37 (17.68)	0.8074	−0.0401
Range	1.11 (1.32)	1.41 (2.89)	0.171	−0.2282
Standard deviation	0.29 (0.39)	0.41 (0.53)	0.1812	−0.2229
180–240 s (MSVC)				
Mean	63.91 (16.16)	61.18 (17.14)	0.7605	−0.0501
Range	0.95 (0.91)	1.51 (1.96)	0.0154*	−0.4217
Standard deviation	0.33 (0.21)	0.41 (0.6)	0.017*	−0.4154

Abbreviations: AG: activation group; CG: Control group; MSVC: maximum sustained voluntary contraction; PC: phasic contraction.

* *p* < 0.05, Mann–Whitney test.

Table 6 shows the intervals in the different groups, contributing to the compression of the infrared spectroscopy during the dynamic responses in the PFM in the transition between rest, PC, and MVSC. The mean oxygenation level in the bulbospongiosus muscle was significantly different among the intervals of PC‐rest (*p* = 0.009, Mann–Whitney test), MVSC‐rest (*p* = 0.015, Mann–Whitney test), and PC‐MVSC (*p* = 0.025, Mann–Whitney test). For the transverse muscle, mean oxygenation significantly affected the variation in the PC‐rest intervals (*p* = 0.023, Mann–Whitney test) and PC‐MVSC (*p* = 0.005, Mann–Whitney test). For the oxygenation amplitude variable, the bulbospongiosus muscle was significantly different between the intervals PC‐rest (*p* = 0.050, Mann–Whitney test) and MCSC‐rest (*p* = 0.001, Mann–Whitney test). For the standard deviation variable, there was a statistically significant difference in the MVSC‐rest interval (*p* = 0.007, Mann–Whitney test) for the bulbospongiosus muscle. No significant differences were found in the oxygenation amplitude or standard deviation in the intervals studied for the transverse muscle.

**TABLE 6:** Analysis of the difference in variation between groups taking into account the evaluation intervals in each muscle.

				Measures of central tendency		Measures of dispersion						
Variables	Muscle	Intervals	Group	Mean	Median	Standard deviation	Min	Max	IQR	Statistic	*p*	Effect size (*r*)
Mean	Bulbospongiosus	PC‐Rest	CG	0.031	0.985	5.164	−18.580	5.780	0.877	242.5	0.009*	−0.0332
			AG	−0.956	−0.510	3.563	−14.090	4.490	2.540			
		MSVC‐Rest	CG	1.028	1.470	4.441	−13.060	8.250	1.860	237.0	0.015*	−0.011
			AG	−1.129	−0.655	5.676	−22.260	7.760	2.020			
		PC‐MSVC	CG	0.996	0.825	1.783	−2.700	5.520	1.375	231.0	0.025*	0.0228
			AG	−0.173	−0.170	2.831	−8.170	7.560	1.005			
Range	Bulbospongiosus	PC‐Rest	CG	−0.707	−0.520	1.204	−3.710	1.280	1.397	98.0	0.050*	0.0261
			AG	1.031	0.130	4.390	−3.550	18.260	1.882			
		MSVC‐Rest	CG	−1.609	−0.685	4.529	−16.650	5.130	1.247	61.0	0.002*	−0.0311
			AG	0.849	0.705	1.877	−3.710	5.310	1.205			
		PC‐MSVC	CG	−0.901	0.025	3.582	−12.940	3.850	1.447	130.0	0.348	−0.0439
			AG	−0.181	0.040	3.390	−12.950	3.840	1.870			
Standard deviation	Bulbospongiosus	PC‐Rest	CG	−0.037	−0.095	0.455	−0.530	1.320	0.445	119.0	0.197	0.0138
			AG	0.302	0.060	1.341	−1.730	5.380	0.460			
		MSVC‐Rest	CG	−0.369	−0.170	0.991	−3.330	1.360	0.492	75.0	0.007*	0.0028
			AG	0.339	0.165	0.841	−1.780	2.190	0.542			
		PC‐MSVC	CG	−0.331	0.030	1.239	−4.650	0.840	0.532	135.0	0.435	−0.0108
			AG	0.037	0.020	0.934	−3.190	1.690	0.597			
Mean	Transverse	PC‐Rest	CG	0.118	0.00	0.484	0.00	2.000	0.000	97.0	0.023*	0.0684
			AG	1.146	1.020	2.033	−2.640	7.290	1.587			
		MSVC‐Rest	CG	2.509	2.430	2.136	−0.140	6.770	2.310	203.0	0.322	0.1554
			AG	1.963	1.145	3.314	−5.090	10.170	3.350			
		PC‐MSVC	CG	2.390	2.430	1.985	−0.140	6.760	2.310	262.0	0.005*	0.0951
			AG	0.816	0.070	2.178	−2.450	8.550	1.715			
Range	Transverse	PC‐Rest	CG	−0.804	−0.390	2.723	−10.160	3.570	1.180	120.0	0.131	−0.0241
			AG	0.195	0.260	2.042	−5.780	3.600	1.640			
		MSVC‐Rest	CG	−1.108	−0.860	3.038	−11.710	2.790	1.160	114.0	0.091	−0.028
			AG	0.388	0.085	2.453	−4.480	8.000	1.575			
		PC‐MSVC	CG	−0.304	−0.210	0.885	−1.860	1.270	1.090	133.5	0.273	−0.003
			AG	0.193	−0.050	1.749	−3.240	4.520	1.645			
Standard deviation	Transverse	PC‐Rest	CG	−0.297	−0.140	1.061	−4.070	1.230	0.310	113.5	0.088	−0.0103
			AG	0.047	0.110	0.676	−1.850	1.480	0.405			
		MSVC‐Rest	CG	−0.400	−0.210	1.171	−4.650	0.980	0.410	127.0	0.195	−0.0129
			AG	0.072	−0.050	0.843	−1.350	2.520	0.410			
		PC‐MSVC	CG	−0.103	−0.080	0.258	−0.580	0.290	0.400	149.0	0.532	−0.0028
			AG	0.024	−0.030	0.610	−1.430	1.430	0.430			

Abbreviations: AG: activation group; CG: Control group; IQR: interquartile range; MSVC: maximum sustained voluntary contraction; PC: phasic contraction.

* *p* < 0.05, Mann–Whitney test.

## 4 Discussion

This study aimed to investigate the behavior of muscle oxygenation variables obtained by an infrared spectroscopy device placed on the skin surface over the muscles of interest under conditions of rest and activation of the pelvic floor muscles of young women. Assessment of these muscles is necessary for treatment [6] and objective measurement of parameters able to identify the effects of therapeutic interventions is recommended [6, 16].

Near‐infrared spectroscopy technology has proven to be an established component in the evaluation of different muscle training interventions, in both healthy and pathological states, to obtain objective information related to the presence and extent of metabolic alterations in response to muscle activation [17, 18, 22, 23, 41].

Although near‐infrared spectroscopy has not yet been used as a surface tool to assess pelvic floor muscles, its feasibility in healthy controls has been previously demonstrated using a consolidated muscle physiology methodology through a transvaginal interface [21, 25]. In the present study, we sought to extend this application to surface assessment, investigating its potential as a complementary method in the analysis of pelvic floor muscles.

To this end, we studied the effects of muscle activation on oxygenation variables obtained by a surface near‐infrared spectroscopy device on the bulbospongiosus and superficial transverse muscles of the perineum. These muscles make up the urogenital diaphragm and the most superficial layer of the pelvic floor [42].

It is believed that although they have muscle fibers with different directions, the activation of the pelvic floor muscles is interdependent and recognized by their mass contraction, moving together like a pelvic belt in a single direction [43].

Obtaining information on superficial muscle oxygenation variability through near‐infrared spectroscopy, although it does not quantify total pelvic floor contractility like other intracavitary devices, expands the methodological options available for clinical evaluation, corroborating the evolution of women's health practices with a view to offering humanized, accessible, and evidence‐based care.

Metrics such as the mean oxygenation, amplitude, and standard deviation were adopted as representative measures of the behavior and variability of muscle oxygenation data [44, 45].

The homogeneity of the sample, consisting of healthy young adult women with no significant structural changes in the pelvic floor and adequate urogynocological status, was fundamental in reducing the influence of confounding factors and ensuring that the differences identified were directly related to the protocol applied, as discussed by Silva et al. [46] and Rosier et al. [47].

This characteristic, combined with methodological rigor, the sensitivity of near‐infrared spectroscopy in detecting metabolic and hemodynamic variations, and the innovative nature of its superficial application to the pelvic floor muscles, consolidates the study's relevant strengths. These aspects reinforce the validity of the findings and support their clinical potential to broaden the understanding of muscle adaptations and contribute to the development of therapeutic strategies in women's health.

The GC, which experienced no active contraction, provided a baseline for muscle oxygenation without interference from metabolic demand and blood flow mechanisms induced by muscle activation. The AG performed the protocol, which included a period of rest followed by progressive muscle activation; it imposed different physiological demands on the muscle tissue, the metabolic and hemodynamic adaptations detectable by near‐infrared spectroscopy applied superficially to the pelvic floor muscles.

The analysis of intragroup behavior revealed a significant increase in mean oxygenation and a reduction in oxygenation amplitude during the test in the CG, a result that differs from the expected physiological behavior and has not been reported in previous near‐infrared spectroscopy protocols, both in pelvic floor muscles and other skeletal muscles [16, 25].

This finding should be interpreted with caution, as it suggests the influence of methodological limitations. Among the factors that may have contributed to this deviation are the way the equipment was fixed, the pressure exerted by the sensor on the tissues, and the duration of the baseline time, which was possibly insufficient to stabilize the variables before collection.

However, it is well known that near‐infrared spectroscopy is an extremely sensitive technique capable of detecting small variations in oxygenation even under resting conditions [18, 48]. The high sensitivity of this technology may have resulted in minor fluctuations in blood flow and oxygen saturation that do not correspond to relevant metabolic and functional changes. In practical terms, the findings can be considered stable or represent normal fluctuations related to the measurement technique.

The skin has sympathetic regulatory mechanisms that act more directly on thermoregulation but indirectly represent a reflex component of the organism that can be influenced by emotional, cognitive, and physiological factors [49, 50, 51]. Furthermore, even superficial touches to the skin, for example, in massage techniques, can cause changes in blood flow and local temperature, interfering with muscle activation, including increased range of motion [52, 53, 54].

Studies on near‐infrared spectroscopy parameters also highlight their relationship with physiological interventions that alter the local circulation of the measurement, such as arterial and venous occlusion or even the use of compressive clothing [18, 55].

In this sense, we suggest that the behavior of the variables observed in the CG may be related to the disturbance caused by coupling the equipment to the structures being assessed. Although not vigorous, we believe that it was able to generate a redistribution of blood flow and local perfusion variability, which showed a pattern of accommodation over time, with a significant reduction in oxygenation amplitude.

The importance of ensuring a stable baseline before data collection is highlighted by our results; regardless of the interface used, it is the achievement of stability before an intervention that guarantees reliable measurements in near‐infrared spectroscopy protocols more than the absolute duration of baseline data collection. Macnab, Stothers, and Deegan [25] describe obtaining analyzable data from 30s as their “arbitrary zero.” In this study, we used the 60s before starting to obtain data. The findings suggest that, with surface monitoring, longer periods of baseline may be necessary to ensure sufficient stability for measurements to be reproducible.

Sousa et al. [56] reported that the magnitude and nature of the adjustment in oxygen consumption at the start of any physical exercise are directly influenced by the intensity of the effort made and allow stratification into moderate, high, and severe.

The comparison between the groups in each interval proposed by the protocol confirmed that the groups had equivalent baseline conditions at muscle rest. However, in the activation intervals, both in PCs and MVSC, the findings revealed significant differences in the variability of the data.

In the bulbospongiosus muscle, the AG showed greater variations in amplitude and standard deviation, indicating metabolic demand during phasic and sustained activation. On the other hand, although the superficial transverse muscle showed less pronounced responses, it also showed significant changes, especially in the interval involving the MVSC.

This finding suggests that near‐infrared spectroscopy not only can identify changes in oxygenation patterns in the pelvic floor muscles in the face of the demands of muscular activation but can also be useful for identifying the relationships between different muscles and the metabolic demands present in each activation.

Previous evidence suggests that near‐infrared data in isometric contractions are more reliable than those from dynamic activation [16]. A study with populations where the intracavitary device was feasible reinforced that MVSC is the most efficient type of muscle activation to obtain information about muscle strength and endurance [25].

For this reason, we expected both muscles to show significant differences in data variability in the MVSC range, which in fact occurred with moderate effect sizes (*r* > 0.40).

However, the particular behavior of the bulbospongiosus in the PC suggests that this muscle has suffered a greater disturbance in oxygenation kinetics in the performance of dynamic activities than the transverse muscle does.

Studies have highlighted the role of bulbospongiosus muscles in rapid and pulsatile contractions that corroborate the final expulsion of urine and contribute to specific moments related to sexual responses [57, 58].

On the other hand, the superficial transverse has shown more tonic and continuous activity, with a fundamental role in stabilizing the perineal body and in structurally supporting the abdomen pressure during exertion and increasing intra‐abdominal pressure [59, 60].

Although there are contributions to the functional aspects of these muscles, a clear and focused approach to the subject has not been found, especially with respect to women's health.

Deepening this knowledge contributes significantly to a better understanding of the performance and role of the pelvic floor muscles in functionally distinct activities. The correlation of these findings with clinical complaints related to women's health could be an important consequence of future studies.

With respect to the impact of activity type on muscle oxygenation, previous studies have shown that oxygen recovery is an important indicator of muscle metabolic capacity [41, 61, 62].

Macnab et al. [25] and Macnab et al. [21], when studying near‐infrared spectroscopy variables on pelvic floor muscles, investigated the half‐recovery time of the hemoglobin difference, a metric that reflects the reoxygenation kinetics of the muscle after an MVSC, and indicated its link with improved muscle metabolic function.

No quantifiable metrics of reoxygenation were observed in this investigation. However, evidence of the dynamic effects of muscle activation was found in the analysis of the transition between intervals.

We observed significant differences between the PC‐rest, MVSC‐rest, and PC‐MVSC intervals in the mean oxygenation of the bulbospongiosus muscle, suggesting that there was a disturbance in oxygenation during contraction, especially in the MVSC, with more pronounced changes in the dynamics of the oxygen supply and greater variability, represented by the significant differences in amplitude and standard deviation for this muscle.

For the transverse muscle, mean oxygenation was the only one that was significantly different between the PC‐rest and PC‐MVSC intervals. This pattern reinforces the evidence that this muscle is more closely related to maintaining prolonged periods of sustained contraction, possibly showing characteristics of greater stability during oxygenation.

However, the fact that the bulbospongiosus muscle appears to be more susceptible to variations induced by muscle activation, while the superficial transverse muscle is more stable, may also be related to the sensitivity of the method in these regions.

Notably, the near‐infrared spectroscopy instrument used in this investigation was not initially designed to be applied to the pelvic floor muscles. The structure attached to its sensors may have hindered data capture and been influenced by the anatomical region in which it had to be positioned.

In this sense, we remain cautious in considering that the differences observed between the muscles evaluated could be attributed to anatomical and functional differences or even the near‐infrared spectroscopy data capture technique in each region.

In fact, MVSC activity was also shown in this analysis to be a more sensitive source of information for near‐infrared spectroscopic data capture. Future studies should take this into account, since it represents more pronounced variations in oxygenation metrics for the different muscles evaluated and may be more useful for investigating the metabolic demands of these muscles.

The equality of the sample limits the applicability of the results to other groups of women and age groups, and the absence of interpretations related to postcontraction reoxygenation also limits the understanding of muscle metabolic capacity. In addition, the accommodation interval, the high sensitivity of near‐infrared spectroscopy, and the fact that the device was adapted for application to the pelvic floor muscles may have influenced fluctuations in the data and not reflected real metabolic changes.

However, even with these limitations, the use of near‐infrared spectroscopy shows promise for identifying metabolic and hemodynamic adaptations in these muscles when applied superficially, reinforcing its great potential for application in the evaluation and development of therapeutic interventions in women's health.

## 5 Limitations

This study has some important limitations that should be considered when interpreting the results.

The near‐infrared spectroscopy equipment is based on processing algorithms that are proprietary to Dynometrics Inc., which may restrict complete transparency regarding oxygenation and hemoglobin calculations.

The way the device is attached to the skin surface may have introduced variations in the pressure exerted and the area effectively evaluated. This variability may influence local blood flow and perfusion, resulting in fluctuations not directly related to muscle metabolic activity.

Initial signal stabilization may require longer periods of tissue and blood flow accommodation. Although the protocol followed previous recommendations, the findings suggest that longer intervals than the baseline may be necessary to ensure greater stability and reproducibility of measurements.

The equipment used was not originally developed for the assessment of pelvic floor muscles. The need for structural adaptation may have limited data capture in some regions and interfered with the sensitivity of the technique.

## 6 Conclusions

Muscle activation caused significant changes in the oxygenation metrics, obtained by surface near‐infrared spectroscopy equipment used to monitor the bulbospongiosus and superficial transverse muscles of the pelvic floor in young adult women, especially during maximum sustained voluntary contraction. Compared with the CG, the AG presented greater variability and dynamic responses, which maintained more stable patterns; however, this variation was possibly influenced by a period of accommodation to the equipment in contact with the surface. The bulbospongiosus muscle showed greater sensitivity induced by muscle activation, whereas the superficial transverse muscle showed more balanced oxygenation patterns, suggesting that anatomical and functional characteristics influenced the response of the bulbospongiosus muscle to the protocol.

The analysis of data variability in AG expands the possibilities for research on pelvic health by allowing the exploration of previously inaccessible patterns of oxygenation and superficial muscle metabolism. Although, unlike transvaginal NIRS, it does not provide quantitative measures of overall pelvic floor function, this resource contributes significantly to the understanding and monitoring of PFM dysfunctions. In addition, it has important clinical advantages, such as greater acceptance and adherence by patients, proving especially useful in sensitive populations or those with contraindications for intracavitary techniques.

## Author Contributions

Conceptualization: Iasmin Pereira Cabral Miranda and João Simão de Melo Neto; methodology, Iasmin Pereira Cabral Miranda and João Simão de Melo Neto. Validation: Iasmin Pereira Cabral Miranda, Pablo Fabiano Moura das Neves, and João Simão de Melo Neto. Formal analysis: Iasmin Pereira Cabral Miranda, Pablo Fabiano Moura das Neves, and João Simão de Melo Neto. Investigation: Iasmin Pereira Cabral Miranda, Pablo Fabiano Moura das Neves, Elizabeth Alves Ferreira, Givago da Silva Souza, and João Simão de Melo Neto. Resources: Iasmin Pereira Cabral Miranda. Data curation: Iasmin Pereira Cabral Miranda, Pablo Fabiano Moura das Neves, Elizabeth Alves Ferreira, Givago da Silva Souza, and João Simão de Melo Neto. Writing – original draft preparation: Iasmin Pereira Cabral Miranda, Marcos Venícius Bentes do Nascimento, Maria Clara Pinheiro do Nascimento, Fernanda Caroline de Jesus Viana, Mayara Carolina Jorge Moraes, Emili Beatriz Chaves de Brito, Fabiane Yasmin de Miranda Lobato, Rayanne Mesquita Bendelack. Writing – review and editing: Iasmin Pereira Cabral Miranda, Marcos Venícius Bentes do Nascimento, Maria Clara Pinheiro do Nascimento, Fernanda Caroline de Jesus Viana, Mayara Carolina Jorge Moraes, Emili Beatriz Chaves de Brito, Fabiane Yasmin de Miranda Lobato, Rayanne Mesquita Bendelack, João Simão de Melo Neto. Visualization: Iasmin Pereira Cabral Miranda, Marcos Venícius Bentes do Nascimento, Maria Clara Pinheiro do Nascimento, Fernanda Caroline de Jesus Viana, Mayara Carolina Jorge Moraes, Emili Beatriz Chaves de Brito, Fabiane Yasmin de Miranda Lobato, Rayanne Mesquita Bendelack. Jorge Moraes, Emili Beatriz Chaves de Brito, Fabiane Yasmin de Miranda Lobato, Rayanne Mesquita Bendelack. Supervision: João Simão de Melo Neto. Project administration: Iasmin Pereira Cabral Miranda, João Simão de Melo Neto. All authors read and agreed with the published version of the manuscript.

## Conflicts of Interest

The authors declare no conflicts of interest.

## Data Availability

The data that support the findings of this study are available from the corresponding author upon reasonable request.

## References

[1] M. Zugaib and R. P. V. Francisco , Zugaib obstetrícia, 3rd ed. (Manole, 2016), 1329.

[2] S. R. Stein , F. V. Pavan , E. F. C. Nunes , and G. F. S. Latorre , “Entendimento da fisioterapia pélvica como opção de tratamento para as disfunções do assoalho pélvico por profissionais de saúde da rede pública,” Revista de Ciências Médicas 27, no. 2 (2018): 65–72, https://docs.bvsalud.org/biblioref/2019/02/980792/med‐2‐00_4242.pdf.

[3] V. G. Miotto , Avaliação da função e do tônus dos músculos do assoalho pélvico em mulheres com e sem constipação intestinal funcional: um estudo observacional transversal [Dissertação] (Universidade de São Paulo, 2022), https://www.teses.usp.br/teses/disponiveis/17/17152/tde‐06052022‐154015/publico/VIVIANEGARNICAMIOTTOco.pdf.

[4] L. F. Iamundo , G. T. A. Nava , P. R. Rocha Júnior , C. B. Prudencio , and A. M. P. Barbosa , “Prevalence and Factors Associated With Pelvic Floor Dysfunction in University Women: A Cross‐Sectional Study,” Fisioterapia e Movimento 35 (2022): e35133, https://www.scielo.br/j/fm/a/9DxPs7pFtmX86SS84vFNHFB/abstract/?format=html&lang=pt.

[5] Associação Brasileira de Fisioterapia em Saúde da Mulher (ABRAFISM) , Recomendações da ABRAFISM sobre Fisioterapia em uroginecologia e coloproctologia em tempos de COVID‐19 (ABRAFISM, 2020), ISBN: 978‐65‐991500‐2‐9, https://abrafism.org.br/wp‐content/uploads/2020/06/Recomendacoes_ABRAFISM_COVID19.pdf.

[6] Y. Abe‐Takahashi , T. Kitta , M. Ouchi , et al., “Confiabilidade e validade da avaliação da força muscular do assoalho pélvico usando o perineômetro MizCure,” BMC Women's Health 20, no. 1 (2020): 257, 10.1186/s12905-020-01127-x. 33213429 PMC7678071

[7] J. B. Silva , Instrumentos de medida para a área de saúde da mulher: significância clínica de questionários e diagnóstico de acurácia da função dos músculos do assoalho pélvico [Tese] (Universidade Federal de São Carlos, 2023), https://repositorio.ufscar.br/bitstream/handle/ufscar/17461/2023_7Mar_Tese_Portugues_JBS.pdf?sequence=1.

[8] R. A. Souza , Construção, validação de conteúdo e de aparência e usabilidade de uma interface virtual destinada à conscientização e relaxamento da musculatura do assoalho pélvico [Dissertação] (Universidade Federal de Pernambuco, 2017), https://repositorioslatinoamericanos.uchile.cl/handle/2250/3979476.

[9] A. T. Silva , Y. P. Silva , and M. P. Furlanetto , “Disfunções do assoalho pélvico em praticantes de Crossfit,” Fisioterapia Brasil 22, no. 2 (2021): 233–248, 10.33233/fb.v22i2.4480.

[10] G. F. S. Latorre , Desenvolvimento de um sistema ideal de telemedicina para aumentar a aderência ao treinamento dos músculos do assoalho pélvico por meio de um aplicativo para smartphone [Tese] (Universidade Federal do Paraná, 2021), https://acervodigital.ufpr.br/handle/1884/73335.

[11] C. S. Saleme , Desenvolvimento de um dispositivo para medir de forma multidirecional a força da musculatura do assoalho pélvico: um estudo piloto [Dissertação] (Universidade Federal de Minas Gerais, 2007), http://hdl.handle.net/1843/SBPS‐7A4HRA.

[12] A. Padoa , L. McLean , M. Morin , and C. Vandyken , “The Overactive Pelvic Floor (OPF) and Sexual Dysfunction. Part 2: Evaluation and Treatment of Sexual Dysfunction in OPF Patients,” Sexual Medicine Reviews 9, no. 1 (2021): 76–92, 10.1016/j.sxmr.2020.04.002. 32631813

[13] B. M. Reis , Função da musculatura do assoalho pélvico em gestantes e puérperas: avaliação manométrica e efeito da massagem perineal [Tese] (Universidade Federal de São Carlos, 2021), https://repositorio.ufscar.br/handle/ufscar/15184.

[14] A. J. Macnab , “The Evolution of Near Infrared Spectroscopy in Urology,” Biomedical Spectroscopy and Imaging 3, no. 4 (2014): 311–344, 10.3233/BSI-140091.

[15] R. A. de Aguiar , T. Turnes , F. K. Borszcz , J. A. G. Raimundo , and F. Caputo , “Near‐Infrared Spectroscopy‐Derived Muscle V̇O_2_ Kinetics After Moderate Running Exercise in Healthy Males: Reliability and Associations With Parameters of Aerobic Fitness,” Experimental Physiology 107 (2022): 476–488, 10.1113/EP090199. 35244956

[16] C. Miranda‐Fuentes , I. M. Guisado‐Requena , P. Delgado‐Floody , et al., “Reliability of Low‐Cost Near‐Infrared Spectroscopy in the Determination of Muscular Oxygen Saturation and Hemoglobin Concentration During Rest, Isometric and Dynamic Strength Activity,” International Journal of Environmental Research and Public Health 17 (2020): 8824, 10.3390/ijerph17238824. 33261036 PMC7730940

[17] M. Tuesta , R. Yáñez‐Sepúlveda , H. Verdugo‐Marchèse , C. Mateluna , and I. Alvear‐Ordenes , “Near‐Infrared Spectroscopy Used to Assess Physiological Muscle Adaptations in Exercise Clinical Trials: A Systematic Review,” Biology 11, no. 7 (2022): 1073, 10.3390/biology11071073. 36101451 PMC9312707

[18] S. Perrey , V. Quaresima , and M. Ferrari , “Muscle Oximetry in Sports Science: An Updated Systematic Review,” Sports Medicine 54 (2024): 975–996, 10.1007/s40279-023-01987-x. 38345731 PMC11052892

[19] B. Lagervaard , J. J. E. Janssen , I. Cuijpers , J. Keijer , V. C. J. de Boer , and A. G. Nieuwenhuizen , “Muscle Mitochondrial Capacity in High‐ and Low‐Fitness Females Using Near‐Infrared Spectroscopy,” Physiological Reports 9, no. 4 (2021): e14838, 10.14814/phy2.14838. 33991439 PMC8123566

[20] A. M. Pilotto , A. Adami , R. Mazzolari , et al., “Near‐Infrared Spectroscopy Estimation of Combined Skeletal Muscle Oxidative Capacity and O_2_ Diffusion Capacity in Humans,” Journal of Physiology 600, no. 18 (2022): 4153–4168, 10.1113/JP283267. 35930524 PMC9481735

[21] A. J. Macnab and L. Stothers , “Measurement of Exercise Treatment Effect From Pelvic Floor Muscle Therapy for Lower Urinary Tract Dysfunction Using Near Infrared Spectroscopy,” Proceedings of SPIE: The International Society for Optical Engineering 11638 (2021): 1–5, 10.1117/12.2586348.

[22] K. B. Bec , J. Grabska , and C. W. Huck , “Near‐Infrared Spectroscopy in Bio‐Applications,” Molecules 25, no. 12 (2020): 2948, 10.3390/molecules25122948. 32604876 PMC7357077

[23] S. Baig , “Healthcare Challenges and Solutions in Pakistan: Utilizing Near‐Infrared Spectroscopy for Clinical and Remote Settings,” Journal of the College of Physicians and Surgeons–Pakistan 33, no. 11 (2023): 1215–1216, 10.29271/jcpsp.2023.11.1215. 37926869

[24] B. Grassi and V. Quaresima , “Near‐Infrared Spectroscopy and Skeletal Muscle Oxidative Function in Vivo in Health and Disease: A Review From an Exercise Physiology Perspective,” Journal of Biomedical Optics 21, no. 9 (2016): 91313, 10.1117/1.JBO.21.9.091313. 27443955

[25] A. J. Macnab , L. Stothers , and E. Deegan , “Development of a Near‐Infrared Spectroscopy Interface Able to Assess Oxygen Recovery Kinetics in the Right and Left Sides of the Pelvic Floor,” Journal of Biomedical Optics 24, no. 7 (2019): 1–5, 10.1117/1.JBO.24.7.075003. PMC6995962 31368259

[26] Gadgets and Wearables , “Humon Hex Review: NIRS Muscle Oxygen Wearable for Athletes,” 2019, https://gadgetsandwearables.com/2019/04/10/humon‐hex‐review/.

[27] HUMON , “Science and Validation,” 2019, https://humon.io/humon‐science‐and‐validation/.

[28] HUMON.ES , “FAQ – Humon Hex,” 2020, https://humon.es/en/faq/.

[29] HUMON.IO , “Humon Hex – Official Website,” 2020, https://humon.io/.

[30] C. J. McManus , S. R. Collier , E. T. Kelley , et al., “Validation of the Humon Hex Muscle Oxygen Monitor,” Medicine & Science in Sports & Exercise 50, no. 6 (2018): 1244–1251, 10.1249/MSS.0000000000001536.

[31] P. Mota , A. Costa , D. Santos , S. Santo , J. Barros , and K. Bø , “Pelvic Floor Muscle Function After Grade II Tears: Surface Electromyography Test‐Retest and Differences Between Nulliparous and Primiparous,” Neurourology and Urodynamics 42, no. 5 (2023): 1162–1168, 10.1002/nau.25180. 37021331

[32] K. Bø , F. Lilleås , T. Talseth , and H. Hedland , “Dynamic MRI of the Pelvic Floor Muscles in an Upright Sitting Position,” Neurourology and Urodynamics 20, no. 2 (2001): 167–174, 10.1002/nau.1004. 11170191

[33] A. Devreese , F. Staes , L. Janssens , F. Penninckx , R. Vereecken , and W. de Weerdt , “Incontinent Women Have Altered Pelvic Floor Muscle Contraction Patterns,” Journal of Urology 178, no. 2 (2007): 558–562, 10.1016/j.juro.2007.03.097. 17570408

[34] S. C. S. Silva , G. C. Reis Junior , S. S. V. Gouveia , and G. P. M. Gouveia , “Análise eletromiográfica e da qualidade de vida na incontinência urinária,” Fisioterapia Brasil 18, no. 5 (2017): 608–615, 10.33233/fb.v18i5.1558.

[35] M. S. Medeiros , Avaliação funcional do assoalho pélvico e atividade física [Trabalho de Conclusão de Curso] (Universidade Estadual da Paraíba, 2013), https://dspace.bc.uepb.edu.br/jspui/bitstream/123456789/5263/1/PDF%20‐%20Marina%20de%20Sousa%20Medeiros.pdf.

[36] E. Moretti , A. G. Moura Filho , J. C. Almeida , C. M. Araújo , and A. Lemos , “Electromyographic Assessment of Women's Pelvic Floor: What Is the Best Place for a Superficial Sensor?,” Neurourology and Urodynamics 36, no. 7 (2017): 1917–1923, 10.1002/nau.23212. 28220534

[37] A. P. Zubreski and A. T. Leonel , “Correlação da autopercepção da atividade MAP e EMG,” Revista Brasileira de Fisioterapia Pelvica 3, no. 1 (2023): 40–50, https://perineo.net/rbfp/3(1)/3(1)40‐50.pdf.

[38] Ł. Oleksy , M. Wojciechowska , A. Mika , et al., “Normative Values for Glazer Protocol in the Evaluation of Pelvic Floor Muscle Bioelectrical Activity,” Medicine (Baltimore) 99, no. 5 (2020): e19060, 10.1097/MD.0000000000019060. 32000454 PMC7004594

[39] J. Cohen , Statistical Power Analysis for the Behavioral Sciences, 2nd ed. (Routledge, 1988).

[40] J. Laycock and D. C. Jerwood , “Pelvic Floor Muscle Assessment: The PERFECT Scheme,” Physiotherapy 87, no. 12 (2001): 631–642, 10.1016/S0031-9406(05)61108-X.

[41] R. Boushel , H. Langberg , J. Olesen , J. Gonzales‐Alonzo , J. Bülow , and M. Kjær , “Monitoring Tissue Oxygen Availability With Near Infrared Spectroscopy (NIRS) in Health and Disease,” Scandinavian Journal of Medicine & Science in Sports 11, no. 4 (2001): 213–222, 10.1034/j.1600-0838.2001.110404.x. 11476426

[42] E. Baracho , Fisioterapia aplicada à saúde da mulher, 5th ed. (Guanabara Koogan, 2012).

[43] T. H. Roza , Prevalência de incontinência urinária feminina e proposta de um protocolo de reabilitação funcional dos músculos do pavimento pélvico para mulheres atletas [Dissertação] (Universidade do Porto, 2011), https://repositorio‐aberto.up.pt/bitstream/10216/56370/2/Dissertao%20de%20MestradoThuane.pdf.

[44] A. M. L. C. Feijoo , “Medidas de dispersão,” in A pesquisa e a estatística na psicologia e na educação [online] (Centro Edelstein de Pesquisas Sociais, 2010), 23–27, http://books.scielo.org.

[45] C. F. S. Rodrigues , F. J. C. Lima , and F. T. Barbosa , “Importância do uso adequado da estatística básica nas pesquisas clínicas,” Revista Brasileira De Anestesiologia 67, no. 6 (2017): 619–625, 10.1016/j.bjan.2017.01.003. 28408080

[46] M. R. Silva , C. Castaño‐García , E. Díaz‐Mohedo , and A. J. Ibáñez‐Vera , “Effects of Activation Protocols on Pelvic Floor Muscle Oxygenation,” Journal of Bodywork and Movement Therapies 24, no. 3 (2020): 307–320, 10.1016/j.jbmt.2020.07.001. 32826005

[47] P. F. W. M. Rosier , “Re: The Article Detrusor Pressures in Urodynamic Studies during Voiding in Women,” International Urogynecology Journal 29, no. 7 (2018): 1071. 29766210 10.1007/s00192-018-3665-8

[48] T. J. Barstow , “Understanding Near Infrared Spectroscopy and Its Application to Skeletal Muscle Research,” Journal of Applied Physiology 126, no. 5 (2019): 1360–1376, 10.1152/japplphysiol.00166.2018. 30844336

[49] V. Donadio , P. Lenzi , P. Montagna , F. Falzone , A. Baruzzi , and R. Liguori , “Habituation of Sympathetic Sudomotor and Vasomotor Skin Responses: Neural and Nonneural Components in Healthy Subjects,” Clinical Neurophysiology 116, no. 10 (2005): 2542–2549, 10.1016/j.clinph.2005.07.009. 16214400

[50] J. L. Greaney , L. M. Alexander , and W. L. Kenney , “Sympathetic Control of Reflex Cutaneous Vasoconstriction in Human Aging,” Journal of Applied Physiology 119, no. 7 (2015): 771–782, 10.1152/japplphysiol.00527.2015. 26272321 PMC4593810

[51] K. Shindo , T. Fukao , N. Kurita , et al., “Sympathetic Outflow to Skin Predicts Central Autonomic Dysfunction in Multiple System Atrophy,” Neurological Sciences 41 (2020): 2241–2248, 10.1007/s10072-020-04340-6. 32198655

[52] U. Chatchawan , K. Jarasrungsichol , and J. Yamauchi , “Immediate Effects of Self‐Thai Foot Massage on Skin Blood Flow, Skin Temperature, and Range of Motion of the Foot and Ankle in Type 2 Diabetic Patients,” Journal of Alternative and Complementary Medicine 26, no. 10 (2020): 1–500, 10.1089/acm.2019.0328. 32349513

[53] L. M. Rodrigues , C. Rocha , H. T. Ferreira , and H. N. Silva , “Lower Limb Massage in Humans Increases Local Perfusion and Impacts Systemic Hemodynamics,” Journal of Applied Physiology (1985) 128, no. 5 (2020): 1217–1226, 10.1152/japplphysiol.00437.2019. 32191595

[54] T. Cagnassi , L. S. Silva , and T. B. Freitas e Silva , “Benefícios da massagem relaxante no corpo e associações com práticas integrativas: revisão de literatura,” Revista Saúde Em Foco 15 (2023): 540–548, https://portal.unisepe.com.br/unifia/wp‐content/uploads/sites/10001/2023/06/Revisa%CC%83o‐de‐artigo‐Beneficios‐da‐massagem‐relaxante.pdf.

[55] A. Lima and J. Bakker , “Espectroscopia no infravermelho próximo para a monitorização da perfusão tecidual,” Revista Brasileira de Terapia Intensiva 23, no. 3 (2011): 341–351, 10.1590/S0103-507X2011000300013. 23949407

[56] A. Sousa , K. Jesus , P. Figueiredo , J. P. Vilas‐Boas , and R. J. Fernandes , “Cinética do consumo de oxigênio nas intensidades de nado moderada e extrema,” Revista Brasileira de Medicina do Esporte 19, no. 3 (2013): 1–5, https://www.scielo.br/j/rbme/a/Sx7HPF3z4kNfMWydryhKM9s/?format=pdf.

[57] A. Zifan , M. Reisert , S. Sinha , et al., “Conectividade dos músculos superficiais do períneo humano: um estudo de tractografia global baseado em imagens de tensor de difusão,” Scientific Reports 8 (2018): 17867, 10.1038/s41598-018-36099-4. 30552351 PMC6294750

[58] D. A. M. C. Bottini , D. V. Silva , R. M. Silva Filho , A. Lúcio , F. Saiki , and A. B. G. S. Pegorare , “Efeitos do treinamento muscular do assoalho pélvico versus ginástica abdominal hipopressiva (GAH) na incontinência urinária de esforço de mulheres climatéricas: ensaio clínico randomizado,” Fisioterapia e Pesquisa 31 (2024): e23000824pt, 10.1590/1809-2950/e23000824pt.

[59] P. Baramée , S. Muro , J. Suriyut , M. Harada , and K. Akita , “Three Muscle Slings of the Pelvic Floor in Women: An Anatomic Study,” Anatomical Science International 95 (2020): 47–53, 10.1007/s12565-019-00492-4. 31165417 PMC6942605

[60] S. Muro and K. Akita , “Pelvic Floor and Perineal Muscles: A Dynamic Coordination Between Skeletal and Smooth Muscles on Pelvic Floor Stabilization,” Anatomical Science International 98, no. 3 (2023): 407–425, 10.1007/s12565-023-00717-7. 36961619 PMC10256674

[61] K. K. McCully , S. Iotti , K. Kendrick , et al., “Simultaneous in Vivo Measurements of HbO_2_ Saturation and PCr Kinetics After Exercise in Normal Humans,” Journal of Applied Physiology (1985) 77, no. 1 (1994): 5–10, 10.1152/jappl.1994.77.1.5. 7961273

[62] S. Jones , K. K. McCully , J. J. Dube , H. J. Green , M. E. Houston , and D. A. Ranney , “Skeletal Muscle Oxidative Metabolism During Exercise Measured With Near‐Infrared Spectroscopy: Reproducibility and Comparison With 31P‐MRS,” Applied Physiology, Nutrition, and Metabolism 41, no. 6 (2016): 572–582, 10.1139/apnm-2015-0518.

